# The RRM domains of PARP14 mediate replication fork degradation in BRCA2-deficient cells

**DOI:** 10.1093/narcan/zcag005

**Published:** 2026-02-11

**Authors:** Anastasia Hale, Katie A Lynch, Ashna Dhoonmoon, Claudia M Nicolae, George-Lucian Moldovan

**Affiliations:** Department of Molecular and Precision Medicine, The Pennsylvania State University College of Medicine, Hershey, PA 17033, United States; Department of Molecular and Precision Medicine, The Pennsylvania State University College of Medicine, Hershey, PA 17033, United States; Department of Molecular and Precision Medicine, The Pennsylvania State University College of Medicine, Hershey, PA 17033, United States; Department of Molecular and Precision Medicine, The Pennsylvania State University College of Medicine, Hershey, PA 17033, United States; Department of Molecular and Precision Medicine, The Pennsylvania State University College of Medicine, Hershey, PA 17033, United States

## Abstract

Degradation of reversed replication forks by nucleases has emerged as a major mechanism of chemosensitivity in BRCA-deficient cells. We previously showed that the mono-ADP-ribosyltransferase PARP14 regulates MRE11 recruitment to reversed replication forks to promote their degradation. This results in genomic instability in BRCA-deficient cells. While it has been shown that PARP14-mediated recruitment of MRE11 to reversed forks promotes their degradation and collapse, how PARP14 binds to nascent DNA is unknown. Here, we show that, in BRCA-deficient cells, PARP14 is recruited to nascent DNA at reversed replication forks via its RRM (RNA Recognition Motifs) domains. We reveal that the RRM domains are necessary for the recruitment of MRE11 to reversed forks to promote nascent strand degradation at stalled replication forks in BRCA2-deficient cells. We also show that these domains are essential for replication stress-induced double-strand break formation in these cells. Our work furthers the understanding of nuclease recruitment and engagement at stalled forks to regulate genomic stability.

## Graphical Abstract

**Graphical Abstract:**
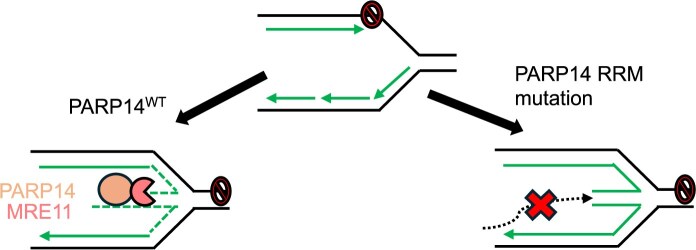

## Introduction

Upon exposure to replication stress, ongoing replication forks decelerate and may arrest [1]. At the site of DNA lesions, replication forks can undergo reversal, a process in which the nascent strands of the sister chromatids anneal. If the forks do not restart and replication fork stalling is prolonged, the fork can collapse, promoting genomic instability [1, 2]. Alternatively, arrested replication forks may be restarted by PrimPol, a primase polymerase [3–5]. In this case, rather than reversing the fork, PrimPol reprimes downstream of the DNA lesion and engages replicative polymerases to continue replication and prevent fork collapse [6].

Collapse of the replication fork commonly occurs in BRCA-deficient cells [7]. The BRCA pathway, an essential component of the homologous recombination (HR) machinery, has also been shown to regulate the resection of DNA by the MRE11 nuclease at reversed forks [8]. The BRCA proteins typically stabilize RAD51 filaments at reversed forks, preventing the engagement of MRE11. However, in cells lacking functional BRCA proteins, the MRE11 nuclease can engage at the unprotected nascent strands, promoting degradation of the fork and subsequent fork collapse [8, 9]. The dynamics of nuclease engagement at reversed replication forks are of particular importance, as protection of stalled forks against nucleolytic degradation is associated with chemoresistance of BRCA-mutant tumors [10].

The BRCA pathway also plays a role in protecting forks restarted via PrimPol repriming [4]. When PrimPol engages at a replication fork, it leaves behind a single-stranded DNA (ssDNA) gap opposite the lesion [3–5]. This gap is expanded bidirectionally by the MRE11 and EXO1 nucleases [11]. Inhibition of the MRE11 exonuclease activity suppresses nascent strand ssDNA gap accumulation in BRCA-deficient cells, suggesting that the MRE11 exonuclease hyper-extends ssDNA gaps if they are not timely filled [12, 13]. Engagement of nucleases in the context of ssDNA gap formation is also of clinical relevance, as accumulation of ssDNA gaps, expanded by nucleases such as MRE11, has been associated with chemosensitivity in BRCA-deficient tumors and has been predicted to underlie the response of BRCA-deficient cells to PARP inhibitors [12, 14]. Overall, through their role in fork degradation and ssDNA gap expansion, the regulation and activity of nucleases such as MRE11 play important roles in determining the sensitivity of BRCA-mutated tumors to genotoxic agents.

We previously showed that the engagement of the MRE11 nuclease at stalled replication forks is primarily regulated by the mono-ADP ribosyl transferase PARP14 [15]. PARP14 is the largest member of the PARP family, consisting of three RNA recognition motif (RRM) domains, three macro domains, a WWE domain, and a PARP catalytic domain [16]. PARP14 has been shown to play a role in the NFKB and JNK pathways, regulate EP4 receptor expression in colon cancer cells, and interact with multiple RNA regulatory proteins [17–19]. Moreover, recent studies have identified PARP14 as essential for genomic stability through the promotion of homologous recombination, and depletion of PARP14 has been shown to increase cellular sensitivity to genotoxic agents [20, 21]. We recently showed that PARP14 recruits MRE11 to reverse replication forks in BRCA-deficient cells, and that loss of PARP14 suppresses MRE11-mediated fork degradation, promoting chemoresistance in these cells [15]. While these studies indicate that PARP14 interacts with stalled replication forks, how PARP14 binds to DNA is currently unknown.

In this study, we reveal that the RRM domains of PARP14 are critical for its recruitment to nascent DNA in cells exposed to replication stress. Moreover, we show that the RRM domains mediate the recruitment of MRE11 to stalled replication forks and promote their degradation in BRCA2-deficient cells. We also reveal that the RRM domains mediate double-stranded DNA break (DSB) formation in these cells, further underlying the role of PARP14 in promoting chemosensitivity via the recruitment of the MRE11 nuclease to unprotected, nascent DNA.

## Materials and methods

### Cell culture and protein techniques

HeLa cells were grown in Dulbecco’s modified Eagle’s media (DMEM). Media was supplemented with 15% FBS and penicillin/streptomycin. HeLa-PARP14^KO^ cells were generated in our laboratory and previously described [15]. To re-express exogenous PARP14 in the knockout cell lines, cells were infected with the lentiviral construct pLV-Puro-SV40 >hPARP14 (VectorBuilder), constitutively expressing PARP14 under the control of the SV40 promoter. After infection, cells were puromycin-selected. Similar vector backbones were used for the PARP14^delRRM1^ (137-end fragment) and PARP14^3FA^ (F54A, F58A, F274A) variants. The mutations in the 3FA variant target conserved residues in the RRM domains, and were selected based on mutations in the RRM domains of RBM45 previously shown to impact ssDNA binding [22].

Gene knockdown was performed using Lipofectamine RNAiMAX. AllStars Negative Control siRNA (Qiagen 1 027 281) was used as a control. The following oligonucleotide sequences (Stealth or SilencerSelect siRNA, ThermoFisher) were used:

BRCA2: GAGAGGCCUGUAAAGACCUUGAAUU.

Denatured whole cell extracts were prepared by boiling cells in 100mM Tris, 4% SDS, 0.5M β-mercaptoethanol. Antibodies used for Western blot, at 1:500 dilution, were:

PARP14: Abcam ab224352

GAPDH: Santa Cruz Biotechnology sc-47724

### DNA fiber combing assays

Cells were incubated with 100 µM IdU and 100 µM CldU as indicated. Drugs (4 mM or 0.4 mM HU, and 200 μM BPA) were added according to the labeling schemes presented. Next, cells were collected and processed using the FiberPrep kit (Genomic Vision EXT-001) according to the manufacturer’s instructions. Samples were added to combing reservoirs containing MES solution (2-(N-morpholino) ethanesulfonic acid), and DNA molecules were stretched onto coverslips (Genomic Vision COV-002-RUO) using the FiberComb Molecular Combing instrument (Genomic Vision MCS-001). Slides were then stained with antibodies detecting CldU (Abcam 6236) and IdU (BD 347580) and incubated with secondary Alexa Fluor 488 (Abcam 150 117) and Cy5 (Abcam 6565) conjugated antibodies. Finally, the cells were mounted onto coverslips and imaged using a confocal microscope (Leica SP5) and analyzed using LASX 3.5.7.23225 software.

### Proximity ligation-based assays

For PLA assays, cells were seeded into 8-chamber slides and 24 h later, were treated with 4 mm HU for 3 h as indicated. Cells were then permeabilized with 0.5% Triton for 10 min at 4°C, washed with PBS, fixed at room temperature with 3% paraformaldehyde in PBS for 10 min, washed again in PBS, and then blocked in Duolink blocking solution (Millipore Sigma DUO82007) for 1 h at 37°C, and incubated overnight at 4°C with primary antibodies. The primary antibodies used were: PARP14 (Abcam ab224352) and MRE11 (Genetex GTX48735). Samples were then subjected to a proximity ligation reaction using the Duolink kit (Millipore Sigma DUO92008) according to the manufacturer’s instructions. Slides were imaged using a confocal microscope (Leica SP5), and images were analyzed using ImageJ 1.53a software. At least 85 cells were quantified for each sample.

For SIRF assays, cells were seeded into 8-chamber slides, and 24 h later, they were pulse-labeled with 50 µM EdU for 10 min or 30 min, followed by drug treatment (4 mM or 0.4 mM HU, and 200 μM BPA) for 1.5 or 3 h as indicated. Cells were permeabilized with 0.5% Triton for 10 min at 4 C, washed with PBS, fixed at room temperature with 3% paraformaldehyde in PBS for 10 min, washed again in PBS, and then blocked in 3% BSA in PBS for 30 min. Cells were then subjected to Click-iT reaction with biotin-azide using the Click-iT Cell Reaction Buffer Kit (ThermoFisher, C10269) for 30 min and incubated overnight at 4 C with primary antibodies diluted in PBS with 1% BSA. The primary antibodies used were: Biotin (mouse: Jackson ImmunoResearch 200–002-211; rabbit: Bethyl Laboratories A150-109A); PARP14 (Abcam ab224352); and MRE11 (Genetex GTX48735). Next, samples were subjected to a proximity ligation reaction using the Duolink kit (Millipore Sigma DUO92008) according to the manufacturer’s instructions. Slides were imaged using a confocal microscope (Leica SP5), and images were analyzed using ImageJ 1.52p software. To account for variation in EdU uptake between samples, for each sample, the number of protein-biotin foci was normalized to the average number of biotin-biotin foci for that respective sample.

### Functional assays

Neutral comet assays were performed using the Comet Assay Kit (Trevigen, 4250–050). Chemical compounds (4mM HU) were added according to the labeling schemes presented. Slides were imaged on a Nikon microscope operating the NIS Elements V1.10.00 software. Olive tail moment was analyzed using CometScore 2.0. Immunofluorescence was performed as previously described [11] using γH2AX (MilliporeSigma JBW301) and 53BP1 (Fortis A300-272A) antibodies. Slides were imaged on a confocal microscope (Leica SP5 STED) and analyzed using ImageJ 1.53a software.

### Fluorescence image acquisition and analysis

Fluorescence images were acquired using a confocal microscope (Leica SP5 STED) with the HC PL APO × 63/1.40 oil objective utilizing the Type F Immersion liquid (11 944 399, Leica Microsystems). The 405-nm laser was used for DAPI (cell nuclei staining) while White Light Laser (WLL) was used for Alexa Fluor Plus 488 (53BP1 staining), Alexa Fluor Plus 568 (γH2AX staining), and Texas Red 594 (SIRF and PLA foci) excitations. HyD detectors were used for a subsequent fluorescence detection. Leica Application Suite X (LAS X) software (Leica Microsystems) was used for optimization of fluorescence detection, signal yields, and spectral unmixing. All images were taken without overexposure at the identical laser intensity, gain, and exposure parameters. The images were saved as 2048 pixels × 2048 pixels, 8-bit multi-channel Leica Image Files (.lif) and exported for quantification to 8-bit TIFF format files using LAS X software. γH2AX, 53BP1, PLA, and SIRF foci were quantified using Image J 1.53a software. Nuclei were outlined via the ‘analyze particles’ function, and a uniform prominence was set using the ‘Find Maxima’ function to detect foci for each antibody. Representative images for figures were prepared using ImageJ and exported as 8-bit TIFF format files. Brightness was linearly adjusted for a given fluorochrome uniformly across entire images and figures to enhance visibility while ensuring the data are accurately represented.

### AlphaFold modeling

Structural models of the PARP14 RNA recognition motif (RRM) domains in complex with single-stranded nucleic acids were generated using the AlphaFold-Server pipeline. Protein sequences corresponding to RRM1 (residues 1–91) and the tandem RRM2–RRM3 construct (residues 146–315) of human PARP14 (UniProt Q8IWY6) were extracted and saved as separate FASTA entries. Short nucleic-acid ligands (6 nt ssRNA or ssDNA oligonucleotides) were designed based on canonical RRM preferences for U/AU-rich motifs; each oligonucleotide was included as an independent chain in the multi-FASTA input. Complexes were predicted using AlphaFold3, which natively supports joint modeling of proteins with nucleic acids. FASTA inputs containing the protein chain(s) and corresponding ssRNA or ssDNA chain were submitted to the AlphaFold Server. Resulting models were ranked using the AlphaFold3 interface and per-residue confidence scores.

### Statistics and reproducibility

For immunofluorescence, SIRF and PLA assays, the *t*-test (two-tailed, unpaired) was used. For DNA fiber assays and comet assays the Mann–Whitney statistical test (two-tailed) was performed. Statistical analyses were performed using GraphPad Prism 10 and Microsoft Excel v2205 software. Statistical significance is indicated for each graph (ns = not significant, for *p* > 0.05; * for *p* ≤ 0.05; ** for *p* ≤ 0.01; *** for *p* ≤ 0.001, **** for *p* ≤ 0.0001)

## Results

### The PARP14 RRM domains regulate the recruitment of PARP14 and MRE11 to stalled replication forks

**Figure 1.**
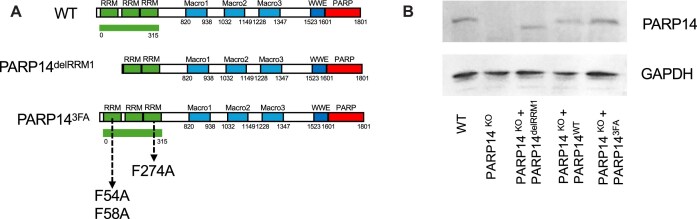
PARP14 domain structure and complementation studies. **(A)** Schematic of the domain architecture of PARP14, including the PARP14 RRM mutants used in this study. **(B)** Western blot showing CRISPR Cas9-mediated knock-out of PARP14 as well as re-introduction of PARP14 variants, including wildtype (WT) PARP14, deletion of the first RRM domain of PARP14 (PARP14^delRRM1^), and 3-point mutations within the RRM domains of PARP14 (PARP14^3FA^).

**Figure 2.**
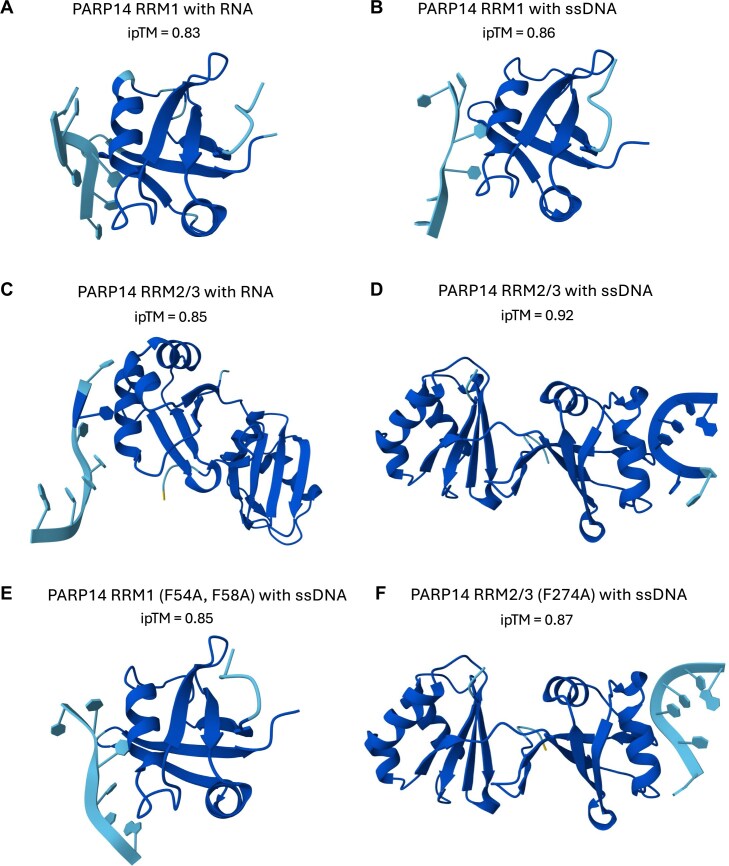
PARP14’s RRM domains are predicted to bind to ssDNA. **(A** and **B)** AlphaFold predicted structure of the RRM1 domain of PARP14 with a 6 nt stretch of RNA **(A)** or ssDNA **(B). (C** and **D)** Alpha-fold predicted structure of the RRM2/3 domains of PARP14 with a 6 nt stretch of RNA **(C)** or ssDNA **(D). (E)** Alpha-fold predicted structure of the RRM1 domain of PARP14 with F54A and F58A point mutations complexed with ssDNA. ipTM (interface predicted Template Modeling) scores generated by AlphaFold are shown. **(F)** AlphaFold predicted structure of the RRM2/3 domains of PARP14 with an F274A point mutation complexed with ssDNA. ipTM values generated by AlphaFold are presented. Values higher than 0.8 represent confident, high-quality predictions, while values below 0.6 suggest a failed prediction.

PARP14 contains three RNA recognition motif (RRM) domains, three Macro domains, a WWE (Tryptophan-Tryptophan-Glutamate) domain and a PARP catalytic domain [16, 23, 24] (Fig. 1). While RRM domains, originally known as RNA recognition motifs, are typically thought to bind to RNA, they have also been shown to be able to bind ssDNA. For example, the RRM1 and RRM2 domains of the RNA-binding protein RBM45 were found to bind ssDNA [22, 25]. To address this, we performed AlphaFold modeling of the RRM domains of PARP14 complexed with short stretches of RNA or ssDNA [26]. The modeling revealed a canonical RRM–nucleic acid configuration with the β-sheet surface oriented toward the nucleic acid (Fig. 2A–D) [27]. These features are characteristic of RRM–nucleic acid interactions and support a direct binding role of the PARP14 RRM domains to ssDNA. Moreover, the predicted binding of RRM1 to ssDNA was associated with a slightly higher confidence in the interaction as compared to RNA (ipTM of 0.86 versus 0.83). RNA and ssDNA also exhibited different patterns of binding to the RRM2/3 domains of PARP14. RNA was predicted to bind with RRM2 of PARP14 with an ipTM of 0.85, while ssDNA exhibited a preference for RRM3 with an iPTM of 0.92.

To determine whether PARP14 binds to DNA via its RRM domains, we created cell lines in which we stably expressed constructs containing wildtype (WT) PARP14, PARP14 with a deleted RRM1 (aminoacids 1–136) domain (PARP14^delRRM1^), and PARP14 with 3 distinct phenylalanine to alanine point mutations (F54A, F58A, F274A) targeting conserved residues within the RRM domains RRM1 and RRM3 (PARP14^3FA^) into HeLa PARP14 knock-out (PARP14^KO^) cells previously created by us [15] (Fig. 1A). These FA mutations were similar to previous mutations made in the RRM domains of RBM45 and shown to abolish ssDNA binding [22, 25]. Modification of the RRM sequence via introduction of the 3FA mutation (F54A, F58A, F274A) appears to alter the predicted binding with ssDNA (Fig. 2E and F). For example, the introduction of the F274A point mutation in RRM3 decreased the iPTM of the structure complexed with ssDNA from 0.92 to 0.87. We validated the re-expression of PARP14 in all these cell lines compared to both parental (WT) and PARP14 knock-out HeLa cells using western blot (Fig. 1B). Expression of all PARP14 variants was comparable to endogenous levels seen in the HeLa WT cells.

**Figure 3.**
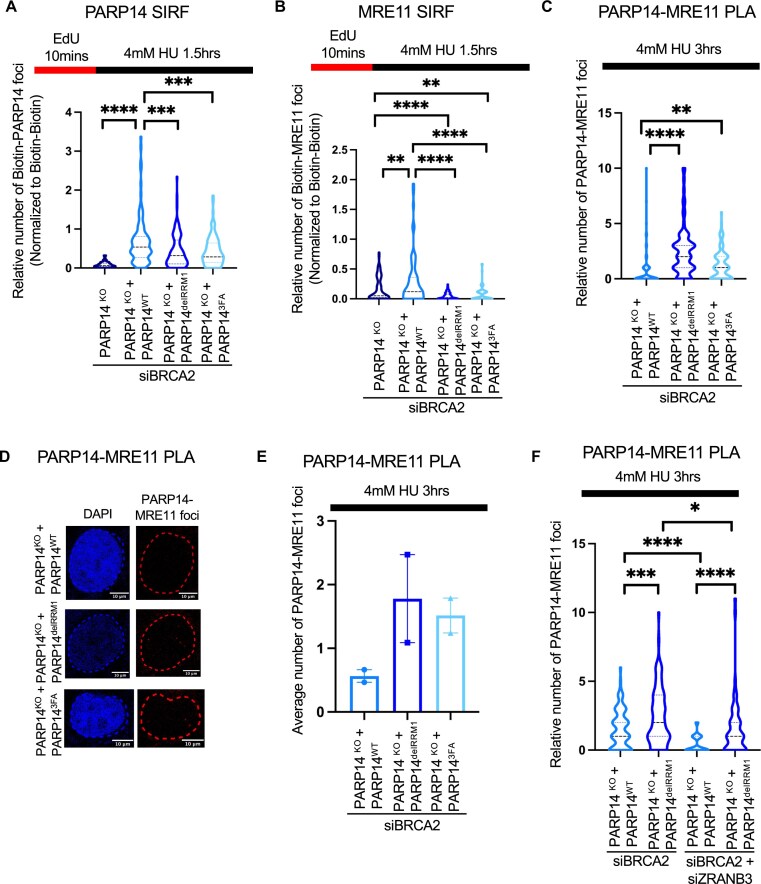
The RRM domains of PARP14 are important for binding to nascent DNA. **(A)** PARP14 SIRF showing that the RRM domains of PARP14 are necessary for recruitment of PARP14 to DNA in BRCA2-depleted cells. At least 96 cells were quantified for each condition. Dotted lines indicate the median values and interquartile range, and asterisks indicate statistical significance (*t*-test, two-tailed, unpaired). Schematic representations of the assay conditions are shown at the top. **(B)** MRE11 SIRF showing that the RRM domains of PARP14 are necessary for recruitment of MRE11 to DNA in BRCA2-depleted cells. At least 63 cells were quantified for each condition. Dotted lines indicate the median values and interquartile range, and asterisks indicate statistical significance (t-test, two-tailed, unpaired). Schematic representations of the assay conditions are shown at the top. **(C, D)** PLA assays showing that disruption of the RRM domains does not impair the interaction between PARP14 and MRE11. **(C)** Representative PARP14-MRE11 PLA experiment. At least 85 cells were quantified for each condition. Dotted lines indicate the median values and interquartile range, and asterisks indicate statistical significance (*t*-test, two-tailed, unpaired). Schematic representations of the assay conditions are shown at the top. **(D)** Representative micrographs of PARP14-MRE11 PLA experiments with scale bars representing 10 µm. **(E)** The means of two independent PARP14-MRE11 PLA experiments are plotted. Dots indicate individual means and bars indicate the range. **(F)** PARP14-MRE11 PLA experiments upon depletion of the fork reversal translocase ZRANB3. At least 56 cells were quantified for each condition. Dotted lines indicate the median values and interquartile range, and asterisks indicate statistical significance (*t*-test, two-tailed, unpaired). Schematic representations of the assay conditions are shown at the top.

We previously showed that PARP14 interacts with nascent DNA at stalled replication forks [15]. To determine whether the RRM domains are responsible for this interaction, we used the *in situ* analysis of proteins at replication forks (SIRF) assay [28], investigating the accumulation of PARP14 at nascent DNA. Since protection of stalled replication forks against nucleolytic degradation is an essential activity of the BRCA pathway in genomic stability, and we previously showed that PARP14 binds the stalled replication forks at increased rates in BRCA-deficient cells [15], we performed this experiment upon siRNA-mediated knockdown of BRCA2. We treated BRCA2-depleted cells with 4 mM hydroxyurea (HU) for 1.5 h to induce fork reversal and degradation. We found that cells expressing PARP14 variants with RRM deletion or mutation exhibited decreased accumulation of PARP14 SIRF foci as compared to cells expressing WT PARP14. These findings suggest that the RRM domains of PARP14 are essential for its recruitment to nascent DNA at reversed forks (Fig. 3A).

We previously showed that PARP14 recruits MRE11 to nascent DNA at reversed replication forks in BRCA-deficient cells [15]. We thus investigated MRE11 recruitment to stalled replication forks in BRCA2-depleted cells, using MRE11 SIRF experiments (Fig. 3B). Similar to the PARP14 SIRF experiment results, cells expressing PARP14 variants with RRM deletion or mutation exhibited decreased accumulation of MRE11 foci as compared to cells expressing WT PARP14. No difference was observed in BRCA-proficient cells (Supplementary Fig. S1). These findings indicate that binding of PARP14 to nascent DNA via its RRM domains is essential for the recruitment of MRE11 to reversed forks.

We previously showed that PARP14 and MRE11 interact with each other [15]. We thus wanted to determine if mutations in the RRM domains of PARP14 affected this interaction. To examine this, we performed proximity ligation assays (PLA) between PARP14 and MRE11 using cells expressing either wild-type PARP14 or PARP14 with mutations in the RRM domains. Surprisingly, we observed an increase in PARP14-MRE11 foci formation in cells expressing the RRM mutants both upon HU treatment and under normal conditions (Fig. 3C–E, Supplementary Fig. S2). Depletion of the fork reversal translocase ZRANB3 suppressed the binding of wildtype PARP14 to MRE11, but the RRM domain mutant PARP14 variant was still able to bind MRE11 under these conditions (Fig. 3F). Altogether, these results indicate that PARP14 binds to nascent DNA at reversed forks via its RRM domains, allowing it to recruit the nuclease MRE11 to the DNA.

### The RRM domains of PARP14 mediate fork degradation and double-strand break formation in BRCA2-deficient cells

**Figure 4.**
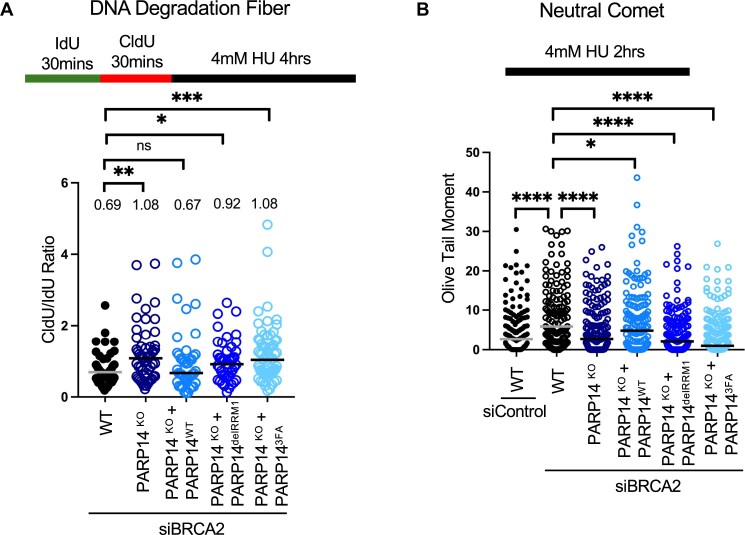
The RRM domains of PARP14 promote fork degradation and DSB formation in BRCA2-depleted cells. **(A)** Fork degradation DNA fiber combing assay showing that loss of PARP14 or mutation of the RRM domains of PARP14 rescues the fork degradation phenotype observed in HR deficient cells. At least 43 fibers were quantified for each condition. Bars indicate the median values, and asterisks indicate statistical significance (Mann–Whitney, two-tailed). Schematic representations of the assay conditions are shown at the top. **(B)** Neutral comet assay showing that loss of PARP14 or mutation of the RRM domains of PARP14 rescues DSB formation in HR-deficient cells. At least 150 cells were quantified for each condition. Bars indicate the median values, and asterisks indicate statistical significance (Mann–Whitney, two-tailed). Schematic representations of the assay conditions are shown at the top.

After determining that PARP14 binds to DNA via its RRM domains, we next sought to determine whether these domains are necessary for the fork degradation phenotype observed in BRCA-deficient cells. As mentioned above, we previously showed that loss of PARP14 suppresses MRE11-mediated nascent strand degradation in BRCA2-deficient cells [15]. To measure fork degradation, we employed the DNA fiber combing assay. We labeled cells for 30 minutes with the thymidine analog IdU, followed by 30 minutes of labeling with CldU, and then treated cells with 4mM hydroxyurea (HU) for 4 hours, a well-established condition that is known to induce fork degradation [8]. If DNA is degraded at reversed forks, the CldU tract will be shortened, leading to a decreased CldU/IdU ratio. As expected, BRCA2 depletion in wild-type HeLa cells caused fork degradation, as shown by a CldU/IdU ratio lower than 1 (Fig. 4A). As previously shown, we observed that knockout of PARP14 rescued the fork degradation observed in these cells. Complementation of the PARP14 knockout cells with wild-type PARP14 restored fork degradation in BRCA2-deficient cells. Interestingly, we observed that cells expressing PARP14 variants with deletion or point mutation in the RRM domains exhibited a rescue of fork degradation. These results indicate that the RRM domains of PARP14 are necessary for its role in promoting fork degradation in BRCA2-deficient cells.

**Figure 5.**
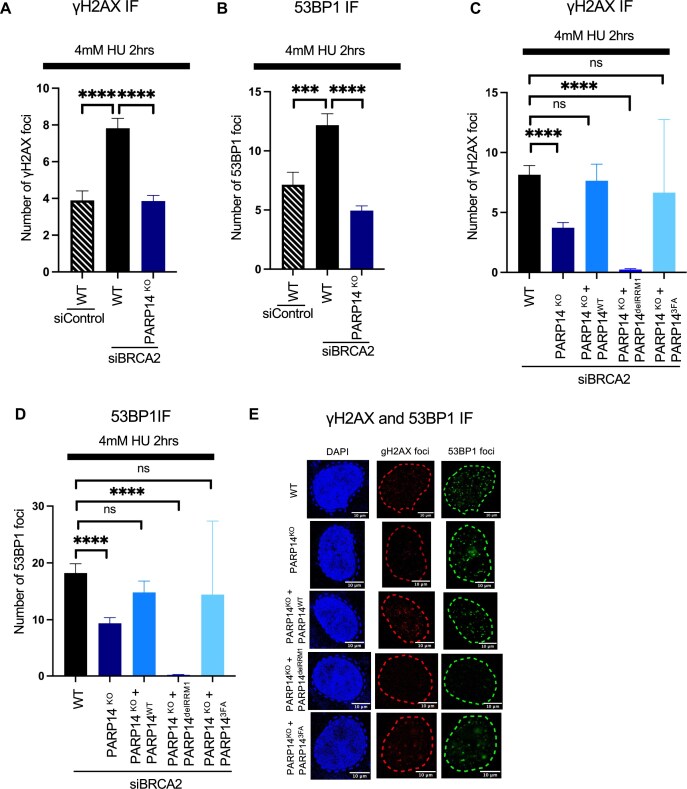
The RRM domains of PARP14 promote DNA damage accumulation in BRCA2-depleted cells. **(A–D)** γH2AX **(A** and **C)** and 53BP1 **(B** and **D)** immunofluorescence showing that loss of PARP14 or mutation of the RRM domains of PARP14 rescues DSB formation in HR-deficient cells. At least 70 cells were quantified for each condition. Bars indicate the mean values, error bars represent the standard error of the mean, and asterisks indicate statistical significance (*t*-test, two-tailed, unpaired). Schematic representations of the assay conditions are shown at the top. **(E)** Representative micrographs with scale bars representing 10 µm.

Next, we investigated whether binding of PARP14 to stalled replication forks via its RRM domains promotes fork collapse and DSB formation observed in BRCA-deficient cells. To determine this, we used the neutral comet assay, in which an increase in the olive tail moment indicates an increase in DSB formation. In agreement with the DNA fiber combing results, loss of PARP14 reduced DSB formation induced by HU treatment in BRCA2-depleted cells, and re-expression of wild-type PARP14 in these cells restored DSB formation (Fig. 4B). In contrast, re-expression of PARP14 variants with deleted or mutated RRM domains did not restore DSB formation, indicating that the RRM domains of PARP14 are necessary for DSB formation in BRCA2-depleted cells after exposure to 4 mM HU. We sought to validate this finding using immunofluorescence (IF) for yH2AX and 53BP1, known markers of DSB formation [29, 30]. We first sought to confirm, using the PARP14^KO^ cell lines, that expression of PARP14 promotes DSB formation in BRCA-deficient cells exposed to HU. We indeed observed that knock-out of PARP14 decreases both yH2AX and 53BP1 foci formation in cells with knock-down of BRCA2 (Fig. 5A and B). We then investigated whether the RRM domains of PARP14 are necessary for this phenomenon. Repeating the yH2AX and 53BP1 immunofluorescence experiments in the PARP14 knockout cell lines re-expressing wild-type or mutated PARP14 RRM domains, we observed that, like PARP14^KO^ cells, cells expressing PARP14 RRM deletion exhibited a decrease in yH2AX and 53BP1 foci formation; in contrast, PARP14 knockout cells re-expressing wild-type PARP14 showed normal induction of yH2AX and 53BP1 foci (Fig. 5C–E). While the RRM deletion variant consistently showed reduced DNA damage induction in both the neutral comet and the yH2AX and 53BP1 immunofluorescence experiments, the 3FA mutant showed a significant reduction in the neutral comet assay, but a much higher variation in the immunofluorescence experiments. It is unclear if this is caused by the different readouts measured by these assays (physical breakage of the DNA for the neutral comet assay as opposed to DNA foci formation by DNA damage signaling and repair proteins) or by a potential residual DNA-binding activity of the 3FA mutant. Overall, these results indicate that binding of PARP14 to nascent DNA at reversed replication forks via its RRM domains promotes their degradation and subsequent collapse, resulting in DSB formation and genomic instability.

## Discussion

**Figure 6.**
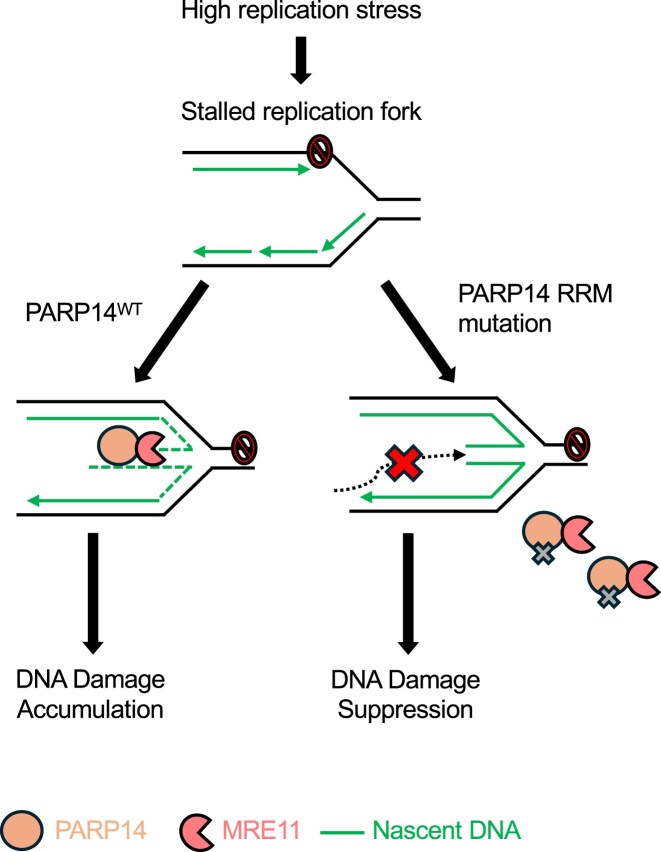
Schematic representation of the proposed model. The RRM domains of PARP14 bind to nascent DNA at stalled replication forks. The binding of PARP14 to the replication fork promotes the recruitment of MRE11, which degrades the fork via its exonuclease activity, promoting chemosensitivity. When the PARP14 is unable to bind to nascent DNA via its RRM domains, an increase in PARP14 binding to MRE11 is observed. This prevents MRE11 from engaging the nascent DNA, preventing fork degradation, and promoting chemoresistance.

Genomic instability, a hallmark of BRCA-deficient cells, underlies BRCA-associated carcinogenesis, but also the sensitivity of BRCA-deficient tumors to genotoxic agents. Because restoration of genome protection is associated with therapeutic resistance of BRCA-deficient tumors, it is essential to understand the mechanisms by which these cells acquire genomic instability [1, 2, 10]. One of the main mechanisms by which BRCA-deficient cells acquire DNA damage that promotes their sensitivity to chemotherapeutic agents is via replication fork stalling and collapse. The MRE11 nuclease, recruited by PARP14, plays a key role in degrading nascent DNA at reversed forks, enhancing genomic instability and chemosensitivity [15]. Our findings reveal that, in BRCA-deficient cells, binding of PARP14 to nascent DNA at reversed forks via its RRM domains is essential for the engagement of MRE11 at unprotected DNA at reversed forks to promote their degradation (Fig. 6).

While PARP14 has been shown to bind to nascent DNA at reversed replication forks and recruit MRE11 to promote degradation of the fork via its exonuclease activity, little is known about how PARP14 binds to DNA [15]. PARP14 contains three RNA recognition motif (RRM) domains, three macro domains, a WWE domain, and a PARP catalytic domain [16]. Though RRM domains are classically thought to bind to RNA, it has been shown that RRM domains have the capacity to bind to ssDNA [25]. For example, a study by Chen et al. investigated the RNA-binding mechanism of the RNA-binding protein RBM45, revealing that both the RRM1 and RRM2 domains of RBM45 recognized the GAC sequence of ssDNA [22]. Our results presented here suggest that PARP14 binds to ssDNA at reversed replication forks via its RRM domains. Loss or mutation of these domains results in not only loss of localization of PARP14 to nascent DNA, but also the rescue of dsDNA break formation and accumulation of genomic instability after exposure to genotoxic agents in BRCA-deficient cells. Moreover, we reveal that binding of PARP14 to nascent DNA via its RRM domains is necessary for the recruitment of MRE11 to reversed replication forks. When MRE11 is not recruited to reversed forks, nascent DNA degradation is halted, preventing the accumulation of dsDNA breaks and subsequent genomic instability. These findings further implicate PARP14 as an essential protein for promoting chemoresistance in BRCA-deficient cells. Future directions could include exploring whether patient mutations in the RRM domains of PARP14 promote their resistance to chemotherapy or cancer recurrence.

Throughout these experiments, we consistently observed a more variable impact of the 3FA point mutant compared to the delRRM1 variant. While the general trend was similar, the 3FA mutant tended to show a higher variation, reflected in higher error bars. This was particularly the case for the DNA damage immunofluorescence experiments, where the 3FA mutant does not show a significant reduction in γH2AX and 53BP1 foci formation, as opposed to the neutral comet assay, where the 3FA mutant displays a reduction in double-strand break formation. We speculate that this trend reflects the possibility that the 3FA point mutant retains DNA binding activity to a greater extent than the deletion mutant, resulting in a milder, less consistent phenotype.

The interaction between PARP14 and MRE11 is crucial for understanding replication fork dynamics, particularly what promotes their degradation, resulting in sensitivity to genotoxic agents. We show here that MRE11 is recruited to nascent DNA upon binding of PARP14 to DNA via its RRM domains. We speculate that, under normal conditions, the PARP14-MRE interaction in the nucleus promotes the recruitment of MRE11 to reversed forks as a complex with PARP14, which binds ssDNA at reversed forks, thus bringing MRE11 in proximity to its DNA substrate. In the PLA experiments, we observed an increase in the interaction between MRE11 and PARP14 upon loss or mutation of the PARP14 RRM domains. We speculate that the decreased presence of MRE11 at reversed forks, due to decreased binding of PARP14, may result in an increase in MRE11 availability, promoting an increase in the interaction between PARP14 and MRE11 off nascent DNA. Moreover, it is unclear as to which domains of MRE11 and PARP14 are responsible for their interaction with one another and whether the availability of the PARP14 RRM domains affects this interaction. It is possible that the mutation or deletion of the RRM domains affects the overall folding of PARP14, resulting in a tighter binding of MRE11. Regardless, the recruitment of MRE11 by binding of PARP14 to nascent DNA via its RRM domains plays a key role in the degradation of reversed replication forks and the promotion of chemosensitivity in BRCA-deficient cells.

## Supplementary Material

zcag005_Supplemental_Files

## Data Availability

The source data underlying all figures are provided with this paper, including the values plotted in graphs, the exact P-values, and the uncropped blots are presented in Supplementary Table S1.
