# Supplementary material for: The RRM domains of PARP14 mediate replication fork degradation in BRCA2-deficient cells
Source: NAR Cancer. 2026 Feb 11;8(1):zcag005. doi: 10.1093/narcan/zcag005 (PMC12891911; doi:10.1093/narcan/zcag005)
Supplement: zcag005_Supplemental_Files [file zcag005_supplemental_files.zip › Supplementary Data.pdf]

Legends to Supplementary Material

The RRM domains of PARP14 mediate replication fork degradation in BRCA2-deficient cells

Hale, Lynch, Dhoonmoon, Nicolae and Moldovan

Legends to Supplementary Tables

Supplementary Table S1. The source data underlying each of the main and supplementary figure panels, including: the values plotted in graphs, the exact p-values, and the uncropped blots.

Legends to Supplementary Figures

Supplementary Figure S1. MRE11 SIRF showing no difference in MRE11 recruitment to nascent DNA in BRCA2-proficient cells. At least 100 cells were quantified for each condition. Bars indicate the mean values, error bars represent standard error of the mean, and asterisks indicate statistical significance (t-test, two-tailed, unpaired). Schematic representations of the assay conditions are shown at the top.

Supplementary Figure S2. PARP14-MRE11 PLA experiment under various conditions. At least 70 cells were quantified for each condition. Dotted lines indicate the median values and interquartile range, and asterisks indicate statistical significance (t-test, two-tailed, unpaired). Schematic representations of the assay conditions are shown at the top.

Figure S1

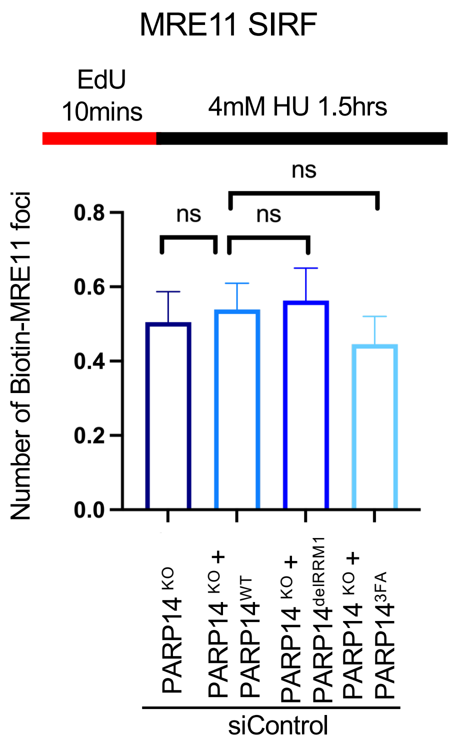
MRE11 SIRF
EdU
10mins
4mM HU 1.5hrs
ns
ns
ns
Number of Biotin-MRE11 foci
0.8
0.6
0.4
0.2
0.0
PARP14 KO
PARP14 KO +
PARP14WT
PARP14 KO +
PARP14delRRM1
PARP14 KO +
PARP143FA
siControl

Figure S2

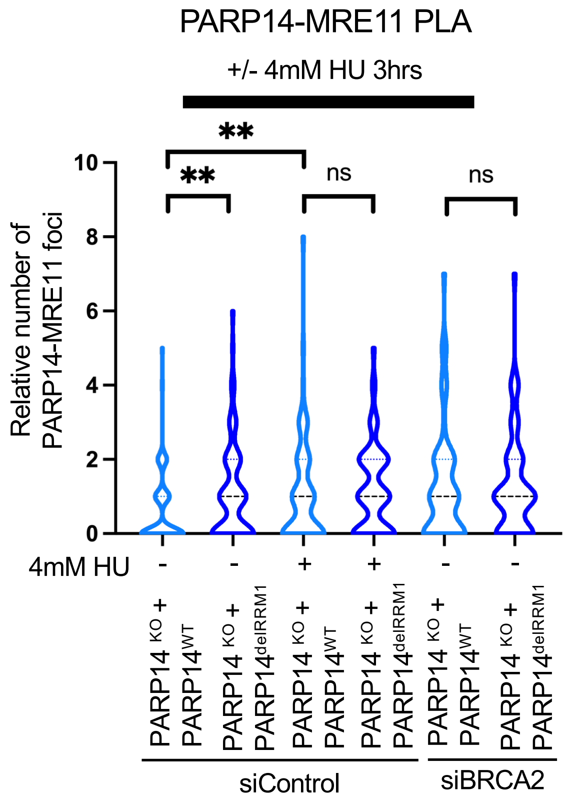
PARP14-MRE11 PLA
+/- 4mM HU 3hrs
**
**
ns
ns
Relative number of PARP14-MRE11 foci
10
8
6
4
2
0
4mM HU
-
-
+
+
-
-
PARP14 KO + PARP14WT
PARP14 KO + PARP14delRRM1
PARP14 KO + PARP14WT
PARP14 KO + PARP14delRRM1
PARP14 KO + PARP14WT
PARP14 KO + PARP14delRRM1
siControl
siBRCA2
